# Factor Structure and Psychometric Properties of the Anxiety Sensitivity Index-3 in an Italian Community Sample

**DOI:** 10.3389/fpsyg.2016.00160

**Published:** 2016-02-16

**Authors:** Marta Ghisi, Gioia Bottesi, Gianmarco Altoè, Enrico Razzetti, Gabriele Melli, Claudio Sica

**Affiliations:** ^1^Department of General Psychology, University of PadovaPadova, Italy; ^2^Department of Developmental and Social Psychology, University of PadovaPadova, Italy; ^3^Institute of Behavioral and Cognitive Psychology and Psychotherapy (IPSICO)Firenze, Italy; ^4^Department of Health Sciences, University of FlorenceFirenze, Italy

**Keywords:** Anxiety Sensitivity Index-3, Italian community sample, factorial structure, bifactor model, psychometric properties

## Abstract

Anxiety Sensitivity (AS) is defined as the fear of anxiety and of arousal-related bodily sensations, arising from erroneous beliefs that these sensations will have adverse consequences. AS plays a key role both in the onset and in the maintenance of several disorders, particularly anxiety disorders. To date, only two studies on American samples have examined the bifactor structure of the Anxiety Sensitivity Index-3 (ASI-3); therefore, findings on different cultures are needed. The main purpose of the present study was to assess the factor structure and psychometric properties of the ASI-3 in an Italian community sample. Participants were recruited from the general population (*N* = 1507). The results of a series of confirmatory factor analyses indicated that the bifactor structure fitted the data better than the most commonly accepted structure for the measure and that it was invariant across gender. Moreover, the current study provided evidence regarding the ASI-3’s reliability and its convergent and divergent validity. Lastly, results pertaining incremental validity of the ASI-3 Physical and Cognitive Concerns subscales above and beyond the total showed that the former was not associated with a measure of physiological anxiety, whereas the latter was weakly associated with a measure of worry. Findings suggest that the ASI-3 is comprised of a dominant general factor and three specific independent factors; given the dominance of the general factor, the use of the ASI-3 total score as a measure of the general fear of anxiety is recommended in both clinical and research settings.

## Introduction

Anxiety Sensitivity (AS) is defined as an individual’s fear of anxiety and of arousal-related bodily sensations (“fear of anxiety symptoms”), including those that may occur during normal physiological processes (e.g., heartbeat acceleration, breathing problems, feeling faint), arising from erroneous beliefs that these sensations will have adverse and harmful physical, psychological, or social consequences such as death, insanity, or social rejection (Reiss and McNally, 1985). In particular, AS is considered a dispositional feature that acts as an anxiety amplifier: When individuals with high levels of AS experience anxiety, they start both catastrophically misinterpreting their introceptive sensations and worrying about them, thus increasing the intensity of anxiety.

AS represents, therefore, a cognitive predisposition to general fearfulness, a vulnerability factor (diathesis) for anxiety psychopathology that plays a central role in both the onset and the maintenance of several anxiety disorders (McNally, 2002; Schmidt et al., 2006; Bernstein and Zvolensky, 2007; Li and Zinbarg, 2007; Calkins et al., 2009; Olatunji and Wolitzky-Taylor, 2009). High AS levels have been found in patients suffering from panic disorder (PD) and agoraphobia (Reiss et al., 1986; White et al., 2006), posttraumatic stress disorder (PTSD; Leen-Feldner et al., 2008; Marshall et al., 2010), social and specific phobias (Sandin et al., 1996; Norton et al., 1997), and obsessive compulsive disorder (Calamari et al., 2008). Typically, patients with PD and/or agoraphobia are characterized by the most elevated AS intensity in comparison to clinical groups with other anxiety pathologies or healthy controls (Deacon and Abramowitz, 2006; Taylor et al., 2007; Mantar et al., 2010; Naragon-Gainey, 2010). For instance, in a recent meta-analysis, Olatunji and Wolitzky-Taylor (2009) reported a large effect size indicative of greater AS among patients with anxiety disorders, especially for those with PD and PTSD, compared to healthy controls (*d* = 1.61 and *d* = 0.71, respectively). Although AS is concurrently and prospectively related to a variety of anxiety disorders, particularly those characterized by hyperarousal (e.g., Ball et al., 1995; Rector et al., 2007; Feldner et al., 2008; Marshall et al., 2010; Schmidt et al., 2010), it has been demonstrated that people with high AS tend to suffer also from non-anxiety psychopathologies such as depression, health anxiety, nicotine, and alcohol addiction, drug problems, chronic pain, and eating disorders (Reiss et al., 1986; Otto et al., 1995; Asmundson, 1999; Stewart et al., 1999; Taylor, 1999; Watt and Stewart, 2000; Anestis et al., 2008; Esteve and Camacho, 2008; Naragon-Gainey, 2010).

The first conceptualizations of AS implicated a unitary construct (Reiss and McNally, 1985; Reiss, 1991; Taylor et al., 1991, 1992). The most widely used self-report measure specifically developed to assess AS is the Anxiety Sensitivity Index (ASI; Reiss et al., 1986; Peterson and Reiss, 1992), a 16-item questionnaire. Although the conceptualization of AS underwent a refinement to include a multidimensional structure, and the original ASI has demonstrated well-established psychometric properties in several samples (Peterson and Plehn, 1999), there was no consensus in the literature regarding its factor structure (Taylor, 1999). Indeed, factor-analytic studies of the original ASI reported results ranging from a one-factor solution (Reiss et al., 1986; Peterson and Heilbronner, 1987; Taylor et al., 1992; Won et al., 1995; Ayvaşɪk, 2000) to 2- to 4-factor solutions (Telch et al., 1989; Wardle et al., 1990; Cox et al., 1996; Zinbarg et al., 1997; Blais et al., 2001; Cho, 2004). Various studies focusing on the latent dimensionality of the original ASI found a hierarchical and multidimensional structure in adult (Zinbarg et al., 1997; Taylor and Cox, 1998a,b) as well as in young individuals (Silverman et al., 2003). In particular, the original ASI was found to be composed of one higher-order factor (general AS) and three lower-order dimensions, namely: Physical Concerns (fear of somatic sensations); Social Concerns (fear of publicly observable anxiety symptoms that may cause social rejection or ridicule); and Cognitive Concerns (fear of cognitive or psychological dyscontrol; Zinbarg et al., 1997). Overall, the most frequently found factor solution was made up of three interrelated factors loading on a single higher-order factor (Taylor, 1999). Potential explanations for the instability in the original ASI factor structure concerned the different factor selection criteria employed across studies and the recruitment of some small samples (Taylor, 1999). Also, since the original ASI was not developed according to a multidimensional model, the Physical Concerns domain was measured by half of the ASI items, leaving only a few items to assess the other two factors (four items for each dimension, generating an unequal item distribution; Taylor and Cox, 1998a; Deacon and Valentiner, 2001). Lastly, the latter two subscales demonstrated weak content validity due to the fact that several items (e.g., “It scares me when I am nauseous”) were not explicitly linked to a specific AS factor (Blais et al., 2001).

Aiming to improve the measure, Taylor and Cox (1998a,b) developed two instruments: the ASI-Revised (ASI-R; Taylor and Cox, 1998a), a 36-item revised scale characterized by the same instructions and response format of the original ASI (10 of 16 original items were incorporated), and the 60-item AS Profile (Taylor and Cox, 1998b). However, studies of both scales demonstrated that their factor solutions were not fully satisfying (e.g., Mohlman and Zinbarg, 2000; Blais et al., 2001; Zvolensky and Forsyth, 2002; Bouvard et al., 2003; Deacon et al., 2003; Olatunji et al., 2005; Deacon and Abramowitz, 2006; Armstrong et al., 2006; Lim et al., 2007; Schmidt et al., 2008). As such, in order to overcome the AS factorial structure instability, Taylor et al. (2007) proposed a new, further-revised multidimensional instrument: the Anxiety Sensitivity Index-3 (ASI-3), which consists of 18 items assessing the three factors most frequently found in previous AS research (six items for each factor): Physical, Social, and Cognitive Concerns. Six items were selected from the ASI-R, whereas five items were derived from the original ASI.

These lower-order facets of AS, consistently assessed with the ASI-3, seem to be differently related to the etiology and maintenance of particular types of anxiety-related disorders. Specifically, Physical Concerns involve fear of physical arousal sensations (e.g., rapid heartbeat) due to concerns that these signal physical illness outcomes or physical catastrophe (e.g., heart attack) and they are strongly associated with panic attacks and PD and/or agoraphobia, health anxiety, and somatization (Taylor et al., 2007; Escocard et al., 2009; Osman et al., 2010; Kemper et al., 2012; Wheaton et al., 2012). Findings pertaining to Social Concerns revealed that this construct significantly correlates with fear of negative evaluation, social phobia and introversion (Taylor et al., 2007; Kemper et al., 2009, 2012; Wheaton et al., 2012; Olthuis et al., 2014). Indeed, they involve fears of publicly observable anxiety symptoms (e.g., shaking) due to concerns that they may result in negative social consequences (e.g., social rejection). Lastly, less clear-cut results have been reported in relation to Cognitive Concerns, which involve fears of cognitive anxiety-related sensations (e.g., difficulty concentrating) due to concerns that they may lead to adverse psychological consequences (e.g., loss of control): many studies did not find a specific relation to any anxiety disorder, but rather to general distress and depression (Taylor and Cox, 1998a; Olthuis et al., 2014), whereas Wheaton et al. (2012) found that they were associated with generalized anxiety disorder.

Taylor et al. (2007) demonstrated that the ASI-3 was characterized by a three-factor hierarchic structure (three subscales for the first-order level and a Global AS factor for the second-order level) both in clinical and non-clinical samples. Such results have been replicated on a mixed sample of anxiety disorder patients and undergraduate students (Wheaton et al., 2012). In contrast, Osman et al. (2010) and Ebesutani et al. (2014) administered the ASI-3 to undergraduate students and found that a bifactor model, consisting of a general factor and three independent orthogonal group factors, added significant improvement over the three-factor hierarchic model in representing the structure of the ASI-3, thus suggesting that the latent structure of the ASI-3 might be unidimensional. Indeed, in bifactor models each item loads on a general factor reflecting what is common among the items and embodies the individual differences on a target dimension (Brown, 2006). Furthermore, two or more orthogonal “group” factors are specified in such a structure: these are specific factors that are orthogonal to one another evaluated by the items capable of explaining item response variance not accounted for by the general factor (Brown, 2006).

It is noteworthy that in the study by Ebesutani et al. (2014) the ASI-3 bifactor model’s results were invariant across gender. As far as psychometric properties are concerned, the ASI-3 subscales proved to be reliable, showing alpha values ranging from 0.73 to 0.91 in the study by Taylor et al. (2007), and from 0.80 to 0.90 (0.93 for the total score) in the study by Wheaton et al. (2012); similarly, Osman et al. (2010) found good values of the composite reliability (i.e., a latent variable modeling procedure to compute true score reliability; range in ρs = 0.80–0.86; ρ = 0.90 for the ASI-3 total). The ASI-3 also showed good convergent, discriminant, and criterion-related validity (Taylor et al., 2007; Osman et al., 2010). Furthermore, it proved to be a better predictor of anxious response to laboratory challenge than the ASI, thus bolstering the improvement of the ASI-3 over the original version (Carter et al., 2009). Importantly, regarding socio-demographic variables, the ASI-3 was revealed to be not affected by age, education level, and gender (Taylor et al., 2007; Osman et al., 2010).

In light of the promising findings mentioned above, it was necessary to verify the stability of the ASI-3 factor structure and its psychometric properties across different cultures and languages. The cross-cultural invariance of the three-factor hierarchic structure has been confirmed by factor analyses in community samples (Sandin et al., 2007; Kemper et al., 2009; Lim and Kim, 2012), as well as in patients with anxiety or mood disorders (Escocard et al., 2009; Mantar et al., 2010; Kemper et al., 2012) from South American, European, Middle Eastern, and Asian countries. Notably, none of these studies tested the bifactor model. Good internal consistency values for the single second-order level (α = 0.91) and for the three first-order level factors (αs = 0.81–0.89) were confirmed in a Brazilian sample made up of patients with anxiety disorders (Escocard et al., 2009), as well as in a Turkish sample consisting of patients with anxiety and mood disorders (single second-order level factor: α = 0.93; three first-order level factors; αs = 0.82–0.89; Mantar et al., 2010). Also, in a Korean college sample, internal consistency values varied from adequate to good (range in αs = 0.73–0.86 and 0.87 for the global scale; Lim and Kim, 2012). Furthermore, moderate associations between the three subscales (range in *rs* = 0.41–0.61) and high correlations between subscales’ scores and the total score (range in *rs* = 0.70–0.86) have been found in this Korean sample (Lim and Kim, 2012). Likewise, in a non-clinical Spanish sample (Sandin et al., 2007) Cronbach’s alpha coefficients ranged from 0.83 to 0.87 (α = 0.91 for the total score); subscales proved to be highly related to the total score (*r*s ranging from 0.80 to 0.83), and moderately inter-correlated (range in *rs* = 0.42–0.59). The 1-month test–retest stability in cross-cultural validations ranged from 0.83 to 0.85 (Sandin et al., 2007) and resulted in 0.64 for the total score (Mantar et al., 2010) in the Spanish and Turkish samples, respectively. Furthermore, patients with anxiety disorders have been successfully distinguished (discriminant validity) from patients with non-anxiety disorders by the ASI-3 in Brazilian, German, and Turkish clinical samples (Escocard et al., 2009; Mantar et al., 2010; Kemper et al., 2012), and it also demonstrated good convergent and divergent validity (Escocard et al., 2009; Mantar et al., 2010; Osman et al., 2010; Kemper et al., 2012; Lim and Kim, 2012).

Lastly, regarding socio-demographic variables, in a sample of Brazilian patients Escocard et al. (2009) replicated findings by Taylor et al. (2007) and Osman et al. (2010), demonstrating that the ASI-3 was not affected by age, education level, and gender. In contrast, females showed higher ASI-3 scores compared to males in a Spanish community sample (Sandin et al., 2007).

### The Current Study

In consideration of the potential utility of the ASI-3, the main aim of the present study was to provide data on its factorial structure and ascertain its reliability, as well as its validity, in an Italian community sample. It is noteworthy that the ASI-3 was designed to be used in both clinical and non-clinical samples. Indeed, given that AS proved to be predictive of panic or anxious responses to challenge and stress (Joiner et al., 2002), a self-report instrument capable of identifying people who are theoretically at risk for developing anxiety disorders or psychological problems is recommended. Furthermore, since some studies outlined the AS’s dimensional structure (Asmundson et al., 2011) and suggested the use of non-clinical samples to evaluate AS because patients often present comorbidity problems (Noyes et al., 2004), studying the ASI-3 in non-clinical samples, in addition to clinical populations, could be useful.

Our first step was to examine the factor structure of the Italian ASI-3 by performing five CFAs that tested five different models. Then the following hypotheses were tested: (1) The ASI-3 factor model would be invariant in terms of gender, as found by Ebesutani et al. (2014); (2) Since few statistics on gender differences have been reported in the literature, we aimed to further investigate them in the present sample. Overall, the results from the few available studies reported no gender differences; therefore, we did not expect differences across gender on the Italian ASI-3 either; (3) The reliability and temporal stability of the ASI-3 would be good; (4) We would find low correlations between the ASI-3 score and age and education in the present adult sample (>18 years); (5) Correlations of the ASI-3 scores with another anxiety measure (convergent validity) would be moderate to high; (6) Correlations of the ASI-3 scores with a measure of depression would be lower than the correlations with a measure of anxiety (divergent validity). Lastly, since Ebesutani et al. (2014) highlighted the need to further examine the incremental utility of the ASI-3 subscales above and beyond the total we also addressed this issue. In particular, we sought to explore whether physical concerns predicted scores on a measure of physiological anxiety above and beyond the general AS factor, and whether cognitive concerns were predictive of scores on a measure of worry above and beyond the general AS factor. We did not formulate any specific hypothesis in regard to social concerns, since no criterion-measures for social anxiety were administered to participants.

## Materials and Methods

### Participants and Procedure

The present sample consisted of 1507 community individuals (38.9% male) from various Italian towns. The current sample represents a subset of a larger sample (*N* = 1617). Participants were identified for the current study based on completion of the ASI-3 with no missing data.^1^ All participants were Caucasian. The mean age of the sample was 38.35 years (*SD* = 14.72; range = 17–80) and the mean years of education was 13.35 (*SD* = 3.24; range = 5–28). Participants listed their marital status as follows: 45.7% single, 47.4% married or cohabitating, 4.8% separated or divorced, 1.6% widowed, and 0.5% other conditions. The employment profile of the total sample was as follows: 44.1% full-time job, 30.2% students, 3.1% part-time job, 4.5% unemployed, 2.6% retired, 2.8% full-time homemaker, and 12.7% other. A subgroup of 80 undergraduate students (40% females; mean age = 27.7 years; *SD* = 5.3) were asked to complete the questionnaires on two occasions 4 weeks apart in order to assess the test–retest reliability of the ASI-3.

The study was carried on in accordance with the Declaration of Helsinki and approved by the institutional review board of the University of Firenze. All participants were recruited on a voluntary basis, by means of a snowball sampling procedure, and gave their written consent before entering the study. The initial recipients were individuals selected and contacted among the acquaintances of several members of our laboratory staff (trainees and post graduate studies born in different regions of Italy). Initial recipients were then invited to select and contact further individuals among the acquaintances. No incentives for participation were given. Eligible participants completed a battery of self-report measures individually administered and rotated in their sequence to control for order effects.

### Materials

All participants completed a background information questionnaire and the following measures:

#### The Anxiety Sensitivity Index-3 (ASI-3; Taylor et al., 2007)

It is an 18-item, self-report measure developed to assess AS. Each item is rated on a 5-point Likert scale ranging from 0 (“not at all”) to 4 (“very much”); the higher the score, the more severe the AS level.

The standard steps outlined in the psychology literature guided the Italian translation process used in the present study (e.g., Brislin, 1986). In the first step, three independent researchers translated the questionnaire from English to Italian and then reached agreement on a common version. Idiomatic Italian at the sixth-grade level was used for this step. Furthermore, the researchers reviewed the common version to ensure that there were no colloquialisms or esoteric sentences that would make interpretations difficult. The shared form was then back-translated by a bilingual individual with extensive knowledge of psychological topics. The back-translation was nearly identical to the original one. As a final step, the Italian ASI-3 items were rated by five experts in anxiety and depressive disorders. Each expert rated the items on a 5-point scale (1 = “not at all,” 5 = “extremely”) for clarity (the extent to which the item is clearly described). The experts’ ratings indicated excellent clarity (mean across all items = 4.4; *SD* = 0.4), indicating that further item refinement was unnecessary.

#### The Beck Anxiety Inventory (BAI; Beck et al., 1988)

It is a 21-item, self-report inventory assessing the severity of anxiety. The Italian version of the BAI was administered to 654 undergraduates, 831 community controls, and 64 anxious patients. Excellent psychometric properties were observed in both the original (internal consistency: α = 0.92; 1-week test–retest reliability: *r* = 0.75 in a community sample) and the Italian version (internal consistency: α = 0.89, α = 0.87, and α = 0.81 in undergraduates, community and anxious patients, respectively; 1-month test–retest reliability: *r* = 0.62 in a student sample; Beck et al., 1988; Sica et al., 2006; Sica and Ghisi, 2007). Good Cronbach’s alpha was also observed in the present study (α = 0.87).

#### The Beck Depression Inventory-II (BDI-II; Beck et al., 1996)

It is a 21-item, self-report scale measuring the severity of affective, cognitive, motivational, vegetative, and psychomotor components of depression. Excellent psychometric properties were observed in both the original (internal consistency: α = 0.92; 1-week test–retest reliability: *r* = 0.75 in a community sample) and the Italian version (internal consistency: α = 0.89, α = 0.87, and α = 0.81 in undergraduates, community and anxious patients, respectively; 1-month test–retest reliability: *r* = 0.62 in a student sample; Beck et al., 1996; Ghisi et al., 2006; Sica and Ghisi, 2007). Internal consistency was also good in the sample employed in the present study (α = 0.85).

#### The Depression Anxiety Stress Scales-21 (DASS-21; Lovibond and Lovibond, 1995)

It consists of 21 items organized into three scales: depression, referring to lack of incentive, low self-esteem, and dysphoria; anxiety, assessing somatic and subjective symptoms of anxiety, as well as acute responses of fear; and stress, measuring irritability, impatience, tension, and persistent arousal (Lovibond and Lovibond, 1995). Findings on the Italian version suggested that use of the total score, measuring a “general distress” factor, could be more appropriate than calculating the three subscale scores separately (Bottesi et al., 2015). The total score of the Italian version showed excellent internal consistency values (α = 0.90 and α = 0.92 in a community and in a mixed clinical sample, respectively), good 2-week test–retest reliability in an undergraduate sample (*r* = 0.74), large convergent/divergent validity coefficients and good criterion-oriented validity (Bottesi et al., 2015). Good Cronbach’s alpha for the total score was also observed in the present study (α = 0.88).

#### The Penn State Worry Questionnaire (PSWQ; Meyer et al., 1990)

It is a 16-item inventory assessing trait worry and, in particular, the generality, excessiveness, and uncontrollability features of pathological worry. The internal consistency of the Italian version of the PSWQ, measured on a community sample was good (α = 0.85; Morani et al., 1999). In the present study, the alpha coefficient was an acceptable 0.70.

### Data Analysis

**FIGURE 1:**
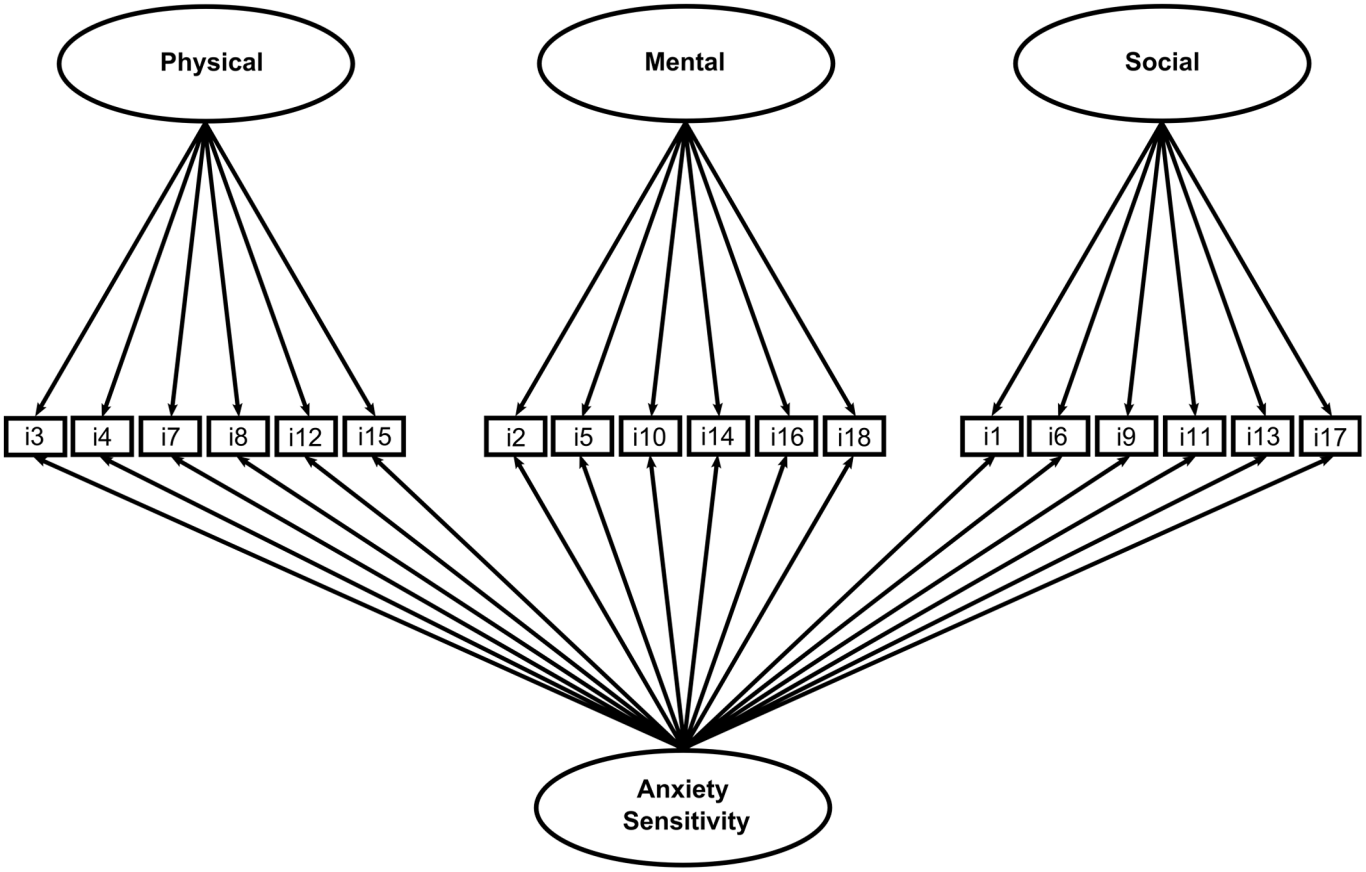
**Factor structure of the ASI-3**.

In order to identify the best factor structure of the ASI-3 in our community sample, and following the recommendations by Reise et al. (2010), we conducted five different confirmatory factor analysis (*CFAs*) that tested five respective theoretical models: (a) a one-dimensional model (all 18 items loading on a single factor); (b) a 2-factor “Physical concerns + Social and Cognitive concerns” correlated traits model; (c) a 2-factor “Physical and Cognitive concerns + Social concerns” correlated traits model; (d) a 3-factor “Physical, Social, and Cognitive concerns” correlated traits model; and (e) a bifactor model in which each of the 18 items is constrained to load on a general factor and on one out of the three (uncorrelated) domain-specific factors (see **Figure 1**). The solutions (b), (c), and (d) were tested since data from the current literature about the contribution of the ASI-3 subscales to the overall internal structure of the questionnaire are inconsistent. Please note that we decided not to test the fit of a second-order model (three dimensions plus a common higher-order AS factor) in light of the fact that this model would have produced identical fit to the three-factor correlated traits model (Brown, 2006).

As suggested by Rhemtulla et al. (2012), given that the data were ordinal and strongly skewed (see **Table 1**), the Weighted Least Squares Mean and Variance (*WLSMV*) robust estimator was employed in all *CFAs*. Assessment of the fit of each model was based on several indices. Since the χ^2^ statistic is extremely sensitive to sample size, two relative fit indices have been considered: the Tucker Lewis index (*TLI*) and the comparative fit index (*CFI*), as they both perform well with small and large samples. For these indices, values >0.95 and >0.97 are associated with acceptable and good fit, respectively (Schermelleh-Engel et al., 2003). The root mean square error of approximation (*RMSEA*) was also used. This is an absolute fit index assessing approximation of parameter estimates to true parameters in the population. *RMSEA* values <0.05 can be considered as a good fit, whereas values between 0.05 and 0.08 as an adequate fit (Schermelleh-Engel et al., 2003). Models were compared according to multiple criteria. First, a qualitative evaluation of the fit indices of each model was considered. Second, the fit of the bifactor model relative to the four competing models was evaluated using scaled χ^2^ difference tests (Satorra, 2000) and the Δ*CFI* criterion (Cheung and Rensvold, 2002). Specifically, if the difference in the *CFIs* between two nested models (Δ*CFI*) is smaller than |0.01|, the hypothesis of no difference in fit between the two competing models should not be rejected and the more parsimonious model should be retained. Given that the scaled χ^2^ difference test is very sensitive to sample size, the interpretation of the results was primarily based on Δ*CFI*. In order to assess reliability, the Omega Hierarchical coefficient^2^ was calculated; standard convention for acceptable reliability is ω > 0.70 (Nunnally, 1978).

**Table 1:** Descriptive statistics for ASI-3 items (*N* = 1507).

Items	Frequency (%)					Mean	*SD*	Skewness
	0	1	2	3	4			
1-Appear nervous	22.2	32.6	27.9	14.1	3.2	1.4	1.1	0.4
2-Going crazy	67.5	22.1	8.4	1.7	0.3	0.5	0.7	1.7
3-Heart beats	37.5	42.8	13.1	6	0.6	0.9	0.9	0.9
4-Stomach upset	78.2	17.3	3.7	0.7	0.1	0.3	0.6	2.3
5-Mind on task	61.0	30.7	6.4	1.5	0.3	0.5	0.7	1.6
6-Tremble	51.8	31,0	12.3	4.2	0.7	0.7	0.9	1.2
7-Breath properly	48.9	35.7	11.9	3.3	0.1	0.7	0.8	1.0
8-Heart attack	50.3	33.2	12.4	3.3	0.8	0.7	0.9	1.2
9-Notice anxiety	47.3	33.2	13.3	5.5	0.7	0.8	0.9	1.1
10-Spaced out	81.5	14.5	3.4	0.6	0.1	0.2	0.5	2.6
11-Blush	50.2	32.8	12.3	3.3	1.3	0.7	0.9	1.3
12-Heart Skipping	60.5	26.6	9.8	2.7	0.5	0.6	0.8	1.5
13-Sweat	57.7	28.2	9.9	3.2	1.0	0.6	0.9	1.5
14-Thoughts speed	87.4	10.3	1.8	0.5	0.0	0.2	0.4	3.3
15-Choke to death	86.5	9.3	3.3	0.8	0.1	0.2	0.5	3.3
16-Thinking clear	69.7	25.2	4.2	0.8	0.1	0.4	0.6	1.7
17-Faint in public	50.2	29.7	12.3	5.9	1.9	0.8	1.0	1.2
18-Mind blank	71.1	21.1	6.0	1.6	0.2	0.4	0.7	1.9

*SD, standard deviation.*

As second step, following the Multi-Group Confirmatory Factor Analysis (MG-CFA) approach of Ebesutani et al. (2014), the measurement invariance of the best ASI-3 factor solution across gender was assessed. We first examined the fit of the single-sample best factor solution within the male and female samples separately. Next, we examined configural invariance across males and females. As configural invariance requires that both genders display the same number of factors as well as identical corresponding items, a baseline model implying the same factorial structure across gender was carried out. In this case, a good-fitting model suggests configural invariance. After testing configural invariance, we constrained item factor loadings and item thresholds to be equal across groups to simultaneously assess metric and scalar invariance (Muthén and Muthén, 2010). In this case, metric and scalar invariance imply that the meaning of the constructs (the factor loadings) and the levels of the underlying items are equal in both groups. Consequently, groups can be compared on their scores on the latent variables. The model implying metric and scalar invariance was evaluated according to 2 criteria: (a) a qualitative analysis of model fit indices and (b) the difference of CFI (Δ*CFI)* against the configural invariance model (Cheung and Rensvold, 2002). A *t*-test comparing males and females on the ASI total score was then performed.

All of the above-mentioned analyses were performed via the open-source software R (R Development Core Team, 2013). For structural equation models, the package *lavaan* (Rosseel, 2012) was used.

Product-moment correlations were performed to evaluate the temporal stability of the ASI-3 scores, as well as convergent and divergent validity, by means of associations between the ASI-3 scores and anxious (BAI score) and depressive (BDI-II score) symptoms. Partial correlations were utilized to establish the specificity of associations when controlling for worry (PSWQ score) and general distress (DASS-21 total score), as well as to test incremental validity of the ASI-3 Physical and Cognitive Concerns subscales (correlations between the ASI-3 Physical and Cognitive Concerns subscales and scores on the BAI and the PSWQ, respectively, controlling for the ASI-3 Total score). Overall, due to the large sample size, the results were interpreted using a significance level of 1% (Simmons et al., 2011).

## Results

### Descriptive Statistics

**Table 1** presents descriptive statistics for the ASI-3 items in the whole sample. Overall, items were strongly skewed in the positive direction (i.e., low frequencies for high values of the ASI-3 scale). It is to note that the mean percentage of non-response per item was.13% (*SD* = 0.12). The three items with the highest percentage of non-response were item 3 (0.40%), item 5 (0.32%), and item 7 (0.26%). In light of these low percentages, it is possible to conclude that none of the ASI-3 items can be considered as problematic.

### Confirmatory Factor Analysis Models

**Table 2** reports fit indices of the bifactor model and all of the competing models. Notably, both the 3-factor correlated traits model and the bifactor model fitted the data well. Nonetheless, the bifactor model resulted in the best factor solution and provided a good fit to observed data [χ^2^(117, *n* = 1507) = 317.7, *p* < 0.001; *CFI* = 0.975; *TLI* = 0.968; *RMSEA* = 0.045]. Scaled χ^2^ difference tests showed that the bifactor model fitted significantly better than the one-dimensional model [χ^2^DIFF(18) = 415.0, *p* < 0.001], the 2-factor (“Physical concerns + Social and Cognitive concerns”) correlated traits model [χ^2^DIFF(17) = 184.0, *p* < 0.001], the 2-factor (“Physical and Cognitive concerns + Social concerns”) correlated traits model [χ^2^DIFF(17) = 237.0, *p* < 0.001], and the 3-factor correlated traits model [χ^2^DIFF(15) = 82.7, *p* < 0.001]. Δ*CFIs* between the bifactor model and the competing models also supported these results (all Δ*CFIs* were larger than 0.022, see **Table 2**).

**Table 2:** Fit statistics for the confirmatory factor analysis models (*N* = 1507).

Model	χ^2^	*df*	*p*	*CFI*	*TLI*	*RMSEA*	Δ*CFI*
Bifactor model	317.61	117	<0.001	0.975	0.968	0.045	-
1-Factor (Unidimensional) model	2298.63	135	<0.001	0.822	0.799	0.112	0.153
2-Factor (Social and Mental + Physical) model	1139.49	134	<0.001	0.915	0.902	0.078	0.060
2-Factor (Physical and Mental + Social) model	1369.36	134	<0.001	0.895	0.880	0.086	0.080
3-Factor model	634.41	132	<0.001	0.953	0.945	0.058	0.022

*CFI, comparative fit index; TLI, Tucker Lewis index; RMSEA, root mean square error of approximation; Δ*CFI*, difference among CFIs between the Bifactor model and the associated competing model.*

As shown in **Table 3**, all loadings associated with the general factor were significant at the 1% level (with all *p*s < 0.001) and had a satisfactory size. Loadings associated with specific group factors were characterized by a generally smaller size. In particular, four loadings associated with the specific group factor Cognitive Concerns were not statistically significant at the 1% level. Notably, **Table 3** also shows that, as emerged in the study by Ebesutani et al. (2014), the decrease in the loadings on the specific factor after accounting for the general factor (i.e., the difference between loadings on the specific factor in the correlated 3-factor model vs. the bifactor model) was greater in the case of the Cognitive Concerns factor.

**Table 3:** The estimated bifactor model (*N* = 1507).

Item	Factor loadings				Proportion of explained variance		
	General	Physical	Mental	Social	by General Factor	by Specific Group Factor	Total
3-Heart beats	0.55	0.43(0.71)			0.30	0.18	0.48
4-Stomach upset	0.57	0.35(0.69)			0.32	0.12	0.44
7-Breath properly	0.64	0.45(0.81)			0.41	0.20	0.61
8-Heart attack	0.49	0.70(0.76)			0.24	0.49	0.73
12-Heart Skipping	0.57	0.60(0.81)			0.32	0.36	0.68
15-Choke to death	0.63	0.31(0.73)			0.40	0.10	0.50
2-Going crazy	0.54		0.57(0.63)		0.29	0.32	0.61
5-Mind on task	0.54		0.60(0.63)		0.29	0.36	0.65
10-Spaced out	0.80		*-0.12*(0.80)		0.64	0.01	0.65
14-Thoughts speed	0.66		*0.06*(0.68)		0.44	0.00	0.44
16-Thinking clear	0.73		*0.09*(0.76)		0.53	0.00	0.53
18-Mind blank	0.68		*0.00*(0.70)		0.46	0.00	0.46
1-Appear nervous	0.26			0.56(0.52)	0.07	0.31	0.38
6-Tremble	0.55			0.50(0.77)	0.30	0.25	0.55
9-Notice anxiety	0.53			0.68(0.82)	0.28	0.46	0.74
11-Blush	0.39			0.47(0.60)	0.15	0.22	0.37
13-Sweat	0.48			0.44(0.67)	0.23	0.19	0.42
17-Faint in public	0.45			0.31(0.58)	0.20	0.01	0.21

*All structural coefficients are standardized. All factor loadings are significant at *p* < 0.01 except for those in italics, for which *p* > 0.01. Loadings of the items on the three correlated factors model are reported in parentheses.*

In terms of explained variance (see Reise et al., 2010), the general factor explained 33% of the total variance, whereas the Physical, Social, and Cognitive Concerns specific factors explained 8, 8, and 4%, respectively, with 47% error. Thus, the general factor accounted for nearly 62% of the common variance extracted. The Omega Hierarchical coefficient for the total score based on our bifactor solution was 0.79. The Omega Hierarchical coefficients for the Physical, Social, and Cognitive Concerns specific factors were 0.36, 0.46, and 0.07, respectively. Taken together, these results support the presence of a relatively strong general ASI-3 factor. In other words, if a composite were formed based on summing the ASI-3 items, we could conclude that 79% of the variance of this composite could be attributable to variance on the general factor (Reise et al., 2010).

### Measurement Invariance of the ASI-3 Bifactor Model Across Gender

Before conducting the MGCFA for ordinal data to evaluate measurement invariance across gender, we examined the distribution of item scores separately for males and females. As for the whole sample (see **Table 1**), the distributions of item scores appeared strongly skewed in both groups. In particular, males did not use the rating “4” in five items (i.e., items 3, 4, 7, 10, and 14) and females did not use the rating “4” in two items (i.e., items 14 and 16; see **Table 1**). Since groups must have the same values on observed variables to perform a multi-group confirmatory analysis with ordinal data, we collapsed scores “3” and “4” into the same category, i.e., “3”.

All steps conducted to verify the measurement invariance of the bifactor model across gender are summarized in **Table 4**. First, single-sample solutions of the ASI-3 bifactor model fitted well in both males (*N* = 582) and females (*N* = 925). Next, our baseline model, which tested to verify the configural invariance of the bifactor model in the whole sample, showed a good fit to the observed data (*CFI* = 0.974, *TLI* = 0.966, *RMSEA* = 0.045). Thus, the same number of factors, reflected by the same set of indicators, was present across gender groups. Our test of equal factor loadings and equal item thresholds also supported invariance of these parameters across males and females, as evidenced by the good fit indices and a Δ*CFI* < 0.01 (see **Table 4**). None of the 18 ASI-3 items were associated with any differential item functioning across gender, allowing for meaningful and interpretable raw score comparisons across males and females.

**Table 4:** Fit statistics for the bifactor model tested for invariance across gender.

Model	*n*	χ^2^	*df*	*p*	*CFI*	*TLI*	*RMSEA*	Δ*CFI*
Males	582	137.42	117	0.096	0.980	0.974	0.039	-
Females	925	254.39	117	<0.001	0.970	0.961	0.047	-
Configural invariance	1507	391.81	234	<0.001	0.974	0.966	0.045	-
Metric/Scalar invariance	1507	542.51	298	<0.001	0.971	0.971	0.041	0.003

*Δ*CFI* = difference among CFIs between configural invariance and Metric/Scalar invariance models.*

In the present sample, a *t*-test comparing males and females on the ASI-3 total score showed a non-significant difference at the 1% level (*M*_Males_ = 9.97, *SD*_Males_ = 7.52, *M*_Females_ = 10.81, *SD*_Females_ = 7.64, *t*(1505) = -2.10, *p* = 0.036). Nonetheless, according to Cohen’s guidelines (Cohen, 1988), a difference between genders is present (higher scores in women than in men) although the magnitude the difference is relatively small (*d*_Cohen_ = -0.11)^3^.

### Association of the ASI-3 Scores with Age and Education

Since, based on the CFAs, ASI-3 resulted in a relatively strong general factor, Pearson’s correlations were calculated only for the ASI-3 total score. In the present sample, neither age (*r* = 0.01) nor education (*r* = -0.03) was associated with the ASI-3 total score (all *p*s > 0.05).

### Temporal Stability, Convergent and Divergent Validity, Incremental Validity

As stated above, Pearson’s correlations were calculated only for the ASI-3 total score. One-month test–retest reliability for the ASI-3 total score was high (Pearson’s *r* = 0.76; *p* < 0.001). In regards to convergent and divergent validities of the ASI-3 total score, it correlated positively and moderately both with the BAI (*r* = 0.41; *p* < 0.001) and the BDI-II (*r* = 0.37; *p* < 0.001). Importantly, only the association with the BAI remained significant after controlling for the PSWQ and for the DASS-21 total score (partial *r* = 0.31; *p* = 0.002), whereas the association with the BDI-II was no longer significant (partial *r* = 0.17; *p* = 0.10).

Lastly, the examination of the incremental utility of the ASI-3 subscales above and beyond the total score of the ASI-3 highlighted that the ASI-3 Physical Concerns subscale was not related to the BAI (*r* = -0.03, *p* = 0.22), whereas the ASI-3 Cognitive Concerns subscale was only weakly related, in terms of effect size, to the PSWQ (*r* = 0.16, *p* < 0.001) after controlling for the general ASI factor.

## Discussion

The ASI-3 was developed to overcome many of the concerns that characterized its previous versions. It has proved to be a valuable and useful comprehensive measure of both clinical and sub-clinical AS symptoms. Indeed, several studies provided support for the ASI-3’s reliability and its convergent, discriminant, criterion-related, and construct validity (Sandin et al., 2007; Taylor et al., 2007; Escocard et al., 2009; Mantar et al., 2010; Osman et al., 2010; Kemper et al., 2012; Lim and Kim, 2012; Wheaton et al., 2012). Nonetheless, inconsistent data about its factor structure has emerged (Sandin et al., 2007; Taylor et al., 2007; Escocard et al., 2009; Kemper et al., 2009, 2012; Mantar et al., 2010; Osman et al., 2010; Lim and Kim, 2012; Ebesutani et al., 2014). As a consequence, the aim of the present study was to assess the factor structure and the psychometric properties of the ASI-3 when applied on a large Italian community sample. Given that environmental factors may vary across cultures, cross-cultural studies in AS manifestations and in instruments devised to assess AS are recommended. Indeed, even though the influence of genetic factors in the etiology of AS has been confirmed (Stein et al., 1999; Taylor et al., 2008), both empirical (Taylor et al., 2008) and retrospective (Stewart et al., 2001; Scher and Stein, 2003) studies have bolstered the role of environmental factors in AS development. For example, although a few studies found that AS is associated in the same way with anxiety and related disorders across socio-cultural contexts (Zvolensky et al., 2001, 2003; Bernstein et al., 2006; Taylor et al., 2007), symptom perception and expression may be affected by cultural variability (Kirmayer et al., 1995). Furthermore, employing a cross-culturally validated instrument allows to compare international research results and to perform international research projects (Van Widenfelt et al., 2005). Finally, the assessment of the ASI-3 factor structure across different cultures represents an important issue because when a measure is used in diverse cultures, it might be interpreted differently (Irvine and Carroll, 1980).

Five CFAs performed on an Italian community sample suggested that, although the original three-factor hierarchic structure (Taylor et al., 2007) evidenced good fit indices, the best factor solution was a bifactor model. Moreover, the general factor accounted for nearly 62% of the common variance extracted, and the Omega Hierarchical coefficient based on our bifactor solution was 0.79, which suggests the presence of a relatively strong general ASI-3 factor. The present findings are consistent with data by Osman et al. (2010) and Ebesutani et al. (2014) and suggest that the ASI-3 consists of a dominant general factor (i.e., general fear of anxiety-related sensations) and three specific, independent, and orthogonal factors. This might mean, as stated by Ebesutani et al. (2014), that “ […] the fear of anxiety in general and the fears of physical, cognitive, and social anxiety-related events appear to be distinct, unrelated fears (once accounting for the general AS factor; Ebesutani et al., 2014, p. 461)” and, consistently with Reise et al. (2010)’s considerations, encourages future research to further explore whether the three AS factors might be independent sub-systems associated to fear event processing. Thus, although the current results are not in contrast to the broadly accepted hierarchical model (i.e., a higher-order factor with three correlated lower-order factors) tested in clinical and community samples (Sandin et al., 2007; Taylor et al., 2007; Escocard et al., 2009; Kemper et al., 2009; Mantar et al., 2010; Lim and Kim, 2012; Wheaton et al., 2012), as a whole our results support the unidimensionality of the latent structure of the ASI-3 and that the general AS factor affects ASI-3 score variation more than the specific anxiety-related fear subscales; therefore, the use of the total score could be more appropriate and informative than calculating the three subscale scores separately. Further support to this argument is that the majority of the loadings associated with the specific Cognitive Concerns factor were not statistically significant (and also the Omega Hierarchical coefficient for the Cognitive Concerns factor was very low), whereas all loadings associated with the general factor were significant at *p* < 0.001 and had a satisfactory size. Interestingly, only two items on the Cognitive Concerns factor in the bifactor model showed satisfying loadings in the current Italian sample, specifically item 2 “When I cannot keep my mind on a task, I worry that I might be going crazy” and item 4 “It scares me when I am unable to keep my mind on a task,” both depicting the interference of anxiety on task execution; in other words, the negative impact of cognitive symptoms is concrete. Therefore, there might be a difference in how cognitive concerns present in the Italian culture as compared to U.S. culture (e.g., Osman et al., 2010; Ebesutani et al., 2014) and that the ASI-3 Cognitive Concerns items may not adequately capture this dimension of AS concerns. Present evidence overall suggest that the ASI-3 can be useful in research and clinical settings, as it represents a cost-effective instrument to evaluate general AS: As a matter of fact, the bifactor model allows a direct exploration of “the extent to which items reflect a common target trait and the extent to which they reflect a primary or subtrait” (Brown, 2006, p. 546).

A further purpose of the present study was to assess measurement invariance of the ASI-3 across gender. In our sample, the bifactor model evidenced equal form, factor loadings, and item thresholds across gender; moreover, none of the 18 ASI-3 items were associated with any differential item functioning across gender. This allowed for meaningful and interpretable raw score comparisons across gender, which prevented potential bias in the score interpretations. These findings confirm previous results from Ebesutani et al. (2014), revealing that scores from the bifactor model are invariant in terms of gender. A comparison analysis demonstrated the existence of small differences between males and females in the AS levels. Therefore, the ASI-3 seems to be quite sensitive to gender differences, in line with findings with a Spanish community sample which showed higher AS levels in women than men (Sandin et al., 2007) but in contrast with a number of other findings in literature which failed in detecting gender differences in the AS levels (e.g., Taylor et al., 2007; Escocard et al., 2009; Osman et al., 2010). From a clinical perspective this could be relevant: indeed, since the prevalence of anxiety disorders varies by gender (*F* > *M*), it is reasonable to hypothesize that AS, which is a factor that contributes to anxiety disorders development, could be higher in women than men. Furthermore, both age and education were not associated with the ASI-3 total score. The present results are in line with findings by Taylor et al. (2007), Escocard et al. (2009), and Osman et al. (2010) and further support the notion that the ASI-3 is insensitive to most socio-demographic variables: overall, these findings are consistent with our initial hypotheses. Mean total scores on the ASI-3 characterizing our community sample (females: *M* = 10.81, *SD* = 7.64; males: *M* = 9.97, *SD* = 7.52) are slightly lower than those observed in other non-clinical samples (e.g., Sandin et al., 2007; Taylor et al., 2007; Mantar et al., 2010; Osman et al., 2010; Lim and Kim, 2012; Wheaton et al., 2012; Ebesutani et al., 2014; mean values for the total score ranged between 10.7 ± 8.1 and 16.74 ± 11.03); to note, all previous studies were conducted on undergraduate samples, whereas our study sample was made up only in part of students (30.2%) and mainly of people from Italian general population.

Regarding psychometric properties, the Omega Hierarchical coefficient for the total score was 0.79, thus suggesting a good reliability for the general factor of the Italian ASI-3 (Nunnally, 1978); these results are consistent with those found by Osman et al. (2010) and Ebesutani et al. (2014). In addition, the 1-month test–retest reliability was good for the ASI-3 total score and in line with results from other cross-cultural studies (Sandin et al., 2007; Mantar et al., 2010). The convergent validity of the Italian version of the ASI-3 was adequate. Overall, the total score evidenced a pattern of specific associations with another anxiety symptom-related measure (i.e., the BAI). Regarding divergent validity, the ASI-3 total score was positively correlated with a measure of depression (i.e., the BDI-II). This result is not surprising, since it is well known that anxiety and depression are characterized by overlapping features and that individuals with depressive symptoms may show high levels of AS (i.e., Taylor et al., 1996; Cox et al., 2001; Armstrong et al., 2006). However, it is noteworthy that the correlations between the ASI-3 and the BAI remained significant even when the effect of worry (assessed by the PSWQ) and general distress (measured by the DASS-21) was controlled; on the other hand, the association between the ASI-3 and the BDI-II did not remain significant when controlling for the effect of worry and general distress after ASI total scores were controlled. Therefore, although there is some shared variability, results from partial correlations highlighted the specificity of the ASI-3 total score, indicating satisfactory convergent and divergent validity. Lastly, an in-depth investigation of the incremental validity of the ASI-3 Physical and Cognitive Concerns subscales above and beyond the total provided only partial support to our hypotheses. Indeed, only the ASI-3 Cognitive Concerns subscale showed an association, albeit weak, with a measure of worry; a similar finding was reported also by Ebesutani et al. (2014). On the other hand, the ASI-3 Physical Concerns subscale did not emerged to be associated with a measure of physiological anxiety. Such results provide further support to the notion that the general AS factor is the more informative score from the ASI-3 as previously observed by Ebesutani et al. (2014).

Some limits of the current study need to be pointed out. First, results from community members were based on a sample that may differ qualitatively from a clinical one; observed findings from the CFA may not be generalizable to patients with anxiety-related disorders and will require thoughtful interpretation. In addition, the recruitment process we employed might not allow one to consider our participants as an accurate representation of the Italian general population. However, we tried to reduce the impact of this limitation by recruiting a large number of participants. A further shortcoming that deserves to be mentioned refers to the small sample size of participants who completed the ASI-3 twice in order to test its temporal stability. Finally, only a few measures were employed to assess convergent, divergent, and incremental validity.

This study represents the first step for studying the bifactor structure of the ASI-3 in the Italian context; next steps will be conducting further studies in clinical Italian samples in order to assess the invariance of the scale structure according to clinical groups and to evaluate how AS is associated with different psychological disorders, as well as to better clarify whether and how the AS subdimensions are related to the general AS dimension. Moreover, additional studies are needed to address the sensitivity of the ASI-3 (as an outcome measure) to treatment effects when applied to Italian patients. Lastly, future study of additional properties of the bifactor model across cultural groups is encouraged.

## Conclusion

The present study sheds light on the factor structure of the ASI-3 and gives robust evidence that the ASI-3 is a brief, reliable, and valid measure to evaluate AS in the Italian population. The present findings support suggestions by Osman et al. (2010) and Ebesutani et al. (2014) to employ the ASI-3 total score to evaluate the general fear of anxiety in both clinical and research settings.

## Author Contributions

MG: Performed literature review and wrote the manuscript. GB: Performed literature review and contributed in writing up the manuscript. GA: Performed statistical analyses. ER: Performed data collection, data entry and support to data analyses. GM: Perfomed data collection. CS: Supervised the entire project.

## Conflict of Interest Statement

The authors declare that the research was conducted in the absence of any commercial or financial relationships that could be construed as a potential conflict of interest.

## Footnotes

1 From the original 1617 participants, 27 (1.7%) participants were excluded due to presence of missing data on each item of the socio-demographic section and on each item of the ASI-3. Next, 49 (3%) participants were excluded due to incomplete values on the socio-demographic section schedule. Of the remaining 1541 participants, only 33 (2%) were excluded due to the presence of at least one missing answer on the ASI-3 items (31 participants with one missing value, and 2 participants with two missing values).

2 The Omega Hierarchical coefficient estimates the proportion of variance in raw scores attributable to a single general trait (i.e., general AS). The extent to which the ASI-3 scores reflect a single general dimension of AS common to all items is expressed by the Total Score Omega Hierarchical coefficient. The degree to which the subscale scores provide reliable variance after accounting for the general factor is expressed by the Omega Hierarchical coefficient for each subscale.

3 The same conclusions hold for the ASI-3 total score on the “collapsed” scale (i.e., 4 points from “0” to “3”): *M*_Males_ = 9.87, *SD*_Males_ = 7.41, *M*_Females_ = 10.67, *SD*_Females_ = 7.46, *t(*1505) = -2.04, *p* = 0.042, *d*_Cohen_ = -0.11.

## References

[B1] Anestis M. D. Holm-Denoma J. M. Gordon K. H. Schmidt N. B. Joiner T. E. (2008). The role of anxiety sensitivity in eating pathology. *Cogn. Ther. Res.* 32 370–385. 10.1007/s10608-006-9085-y

[B2] Armstrong K. A. Khawaja N. G. Oei T. P. S. (2006). Confirmatory factor analysis and psychometric properties of the anxiety sensitivity index - revised in clinical and normative populations. *Eur. J. Psychol. Assess.* 22 116–125. 10.1027/1015-5759.22.2.116

[B3] Asmundson G. J. G. (1999). “Anxiety sensitivity and chronic pain: empirical findings, clinical implications, and future directions,” in *Anxiety Sensitivity: Theory, Research and Treatment of the Fear of Anxiety* ed. Taylor S. (Mahwah, NJ: Erlbaum) 269–285.

[B4] Asmundson G. J. G. Weeks J. W. Carleton N. R. Thibodeau M. A. Fetzner M. G. (2011). Revisiting the latent structure of the anxiety sensitivity construct: more evidence of dimensionality. *J. Anxiety Disord.* 25 138–147. 10.1016/j.janxdis.2010.08.013 20888185

[B5] Ayvaşɪk H. B. (2000). Kaygɪ duyarlɪǧɪ indeksi: geçerlik ve güvenirlik çalışması. *Türk. Psikol. Derg.* 15 43–57.

[B6] Ball S. G. Otto M. W. Pollack M. H. Uccello R. Rosenbaum J. F. (1995). Differentiating social phobia and panic disorder: a test of core beliefs. *Cogn. Ther. Res.* 19 473–482. 10.1007/BF02230413

[B7] Beck A. T. Epstein N. Brown G. Steer R. A. (1988). An inventory for measuring clinical anxiety: psychometric properties. *J. Consult. Clin. Psychol.* 56 893–897. 10.1037/0022-006X.56.6.893 3204199

[B8] Beck A. T. Steer R. A. Brown G. K. (1996). *Manual for the Beck Depression Inventory-II.* San Antonio, TX: The Psychological Corporation.

[B9] Bernstein A. Zvolensky M. J. (2007). Anxiety sensitivity: selective review of promising research and future directions. *Exp. Rev. Neurother.* 7 97–101. 10.1586/14737175.7.2.97 17286543

[B10] Bernstein A. Zvolensky M. J. Kotov R. Arrindell W. A. Taylor S. Sandin B. (2006). Taxonicity of anxiety sensitivity: a multi-national analysis. *J. Anxiety Disord.* 20 1–22. 10.1016/j.janxdis.2004.11.006 16325111

[B11] Blais M. A. Otto M. W. Zucker B. G. McNally R. J. Schmidt N. B. Fava M. (2001). The anxiety sensitivity index: item analysis and suggestions for refinement. *J. Pers. Assess.* 77 272–294. 10.1207/S15327752JPA7702_10 11693859

[B12] Bottesi G. Ghisi M. Altoè G. Conforti E. Melli G. Sica C. (2015). The italian version of the depression anxiety stress scales-21: factor structure and psychometric properties on community and clinical samples. *Compr. Psychiatry* 60 170–181. 10.1016/j.comppsych.2015.04.005 25933937

[B13] Bouvard M. Ayxères-Vighetto A. Dupont H. Aupetit J. Portalier S. Arrindell W. (2003). [Preliminary validation of the French translation of anxiety sensibility index-revised (ASI-R)]. *L’Encéphale* 29 157–164. 14567167

[B14] Brislin R. W. (1986). “The wording and translation of research instruments,” in *Field Methods in Cross-Cultural Research. Cross-Cultural Research and Methodology Series* eds Lonner W. J. Berry J. W. (Thousand Oaks, CA: Sage Publications, Inc.) 137–164.

[B15] Brown T. A. (2006). *Confirmatory Factor Analysis for Applied Research.* New York, NY: Guilford Press.

[B16] Calamari J. E. Rector N. A. Woodard J. L. Cohen R. J. Chik H. M. (2008). Anxiety sensitivity and obsessive-compulsive disorder. *Assessment* 15 351–363. 10.1177/1073191107312611 18310595

[B17] Calkins A. W. Otto M. W. Cohen L. S. Soares C. N. Vitonis A. F. Hearon B. A. (2009). Psychosocial predictors of the onset of anxiety disorders in women: results from a prospective 3-year longitudinal study. *J. Anxiety Disord.* 23 1165–1169. 10.1016/j.janxdis.2009.07.022 19699609 PMC2760601

[B18] Carter M. M. Sbrocco T. Ayati F. (2009). Predicting anxious response to a social challenge and hyperventilation: comparison of the ASI and ASI-3. *J. Behav. Ther. Exp. Psychiatry* 40 434–442. 10.1016/j.jbtep.2009.05.001 19501813

[B19] Cheung G. W. Rensvold R. B. (2002). Evaluating goodness-of-fit indexes for testing measurement invariance. *Struct. Equ. Model. Multidiscip. J.* 9 233–255. 10.1207/S15328007SEM0902_5

[B20] Cho Y. R. (2004). Factor structure of the korean version of the anxiety sensitivity index: confirmatory evidence for a hierarchical model. *Korean J. Clin. Psychol.* 23 207–220.

[B21] Cohen J. (1988). *Statistical Power Analysis for the Behavioral Sciences.* 2nd Edn. Hillsdale, NJ: Erlbaum.

[B22] Cox B. J. Enns M. W. Taylor S. (2001). The effect of rumination as a mediator of elevated anxiety sensitivity in major depression. *Cogn. Ther. Res.* 25 525–534. 10.1023/A:1005580518671

[B23] Cox B. J. Parker J. D. A. Swinson R. P. (1996). Anxiety sensitivity: confirmatory evidence for a multidimensional construct. *Behav. Res. Ther.* 34 591–598. 10.1016/0005-7967(96)00006-X 8826766

[B24] Deacon B. Abramowitz J. (2006). Anxiety sensitivity and its dimensions across the anxiety disorders. *J. Anxiety Disord.* 20 837–857. 10.1016/j.janxdis.2006.01.003 16466904

[B25] Deacon B. J. Abramowitz J. S. Woods C. M. Tolin D. F. (2003). The anxiety sensitivity index - revised: psychometric properties and factor structure in two nonclinical samples. *Behav. Res. Ther.* 41 1427–1449. 10.1016/S0005-7967(03)00065-2 14583412

[B26] Deacon B. J. Valentiner D. P. (2001). Dimensions of anxiety sensitivity and their relationship to nonclinical panic. *J. Psychopathol. Behav. Assess.* 23 25–33. 10.1023/A:1011087322899

[B27] Ebesutani C. McLeish A. C. Luberto C. M. Young J. Maack D. J. (2014). A bifactor model of anxiety sensitivity: analysis of the anxiety sensitivity index-3. *J. Psychopathol. Behav. Assess.* 36 452–464. 10.1007/s10862-013-9400-3

[B28] Escocard M. R. P. G. Fioravanti-Bastos A. C. M. Landeira-Fernandez J. (2009). Anxiety sensitivity factor structure among brazilian patients with anxiety disorders. *J. Psychopathol. Behav. Assess.* 31 246–255. 10.1007/s10862-008-9103-3

[B29] Esteve M. R. Camacho L. (2008). Anxiety sensitivity, body vigilance and fear of pain. *Behav. Res. Ther.* 46 715–727. 10.1016/j.brat.2008.02.012 18396262

[B30] Feldner M. T. Zvolensky M. J. Schmidt N. B. Smith R. C. (2008). A prospective test of anxiety sensitivity as a moderator of the relation between gender and posttraumatic symptom maintenance among high anxiety sensitive young adults. *Depress. Anxiety* 25 190–199. 10.1002/da.20281 17340601

[B31] Ghisi M. Flebus G. B. Montano A. Sanavio E. Sica C. (2006). *Beck Depression Inventory-II. Manuale.* Firenze: Organizzazioni Speciali.

[B32] Irvine S. H. Carroll W. K. (1980). “Testing and assessment across cultures,” in *Handbook of Cross-Cultural Psychology* eds Triandis H. C. Berry J. W. (Boston, MA: Allyn& Bacon).

[B33] Joiner T. E. Schmidt N. B. Schmidt K. L. Laurent J. Catanzaro S. J. Perez M. (2002). Anxiety sensitivity as a specific and unique marker of anxious symptoms in youth psychiatric inpatients. *J. Abnorm. Child Psychol.* 30 167–175. 10.1023/A:1014757300294 12008656

[B34] Kemper C. J. Lutz J. Bähr T. Rddel H. Hock M. (2012). Construct validity of the anxiety sensitivity index–3 in clinical samples. *Assessment* 19 89–100. 10.1177/1073191111429389 22156717

[B35] Kemper C. J. Ziegler M. Taylor S. (2009). Überprüfung der psychometrischen qualität der deutschen version des angstsensitivitätsindex-3. *Diagnostica* 55 223–233. 10.1026/0012-1924.55.4.223

[B36] Kirmayer L. J. Young A. Hayton B. C. (1995). The cultural context of anxiety disorders. *Psychiatr. Clin. North Am.* 18 503–521. 8545264

[B37] Leen-Feldner E. W. Feldner M. T. Reardon L. E. Babson K. A. Dixon L. (2008). Anxiety sensitivity and posttraumatic stress among traumatic event-exposed youth. *Behav. Res. Ther.* 46 548–556. 10.1016/j.brat.2008.01.014 18328463 PMC2362392

[B38] Li W. Zinbarg R. E. (2007). Anxiety sensitivity and panic attacks: a 1-year longitudinal study. *Behav. Modif.* 31 145–161. 10.1177/0145445506296969 17307932

[B39] Lim Y.-J. Kim J.-H. (2012). Korean anxiety sensitivity index-3: its factor structure, reliability, and validity in non-clinical samples. *Psychiatry Investig.* 9 45–53. 10.4306/pi.2012.9.1.45 PMC3285740 22396684

[B40] Lim Y.-J. Yu B.-H. Kim J.-H. (2007). Korean anxiety sensitivity index—revised: its factor structure, reliability, and validity in clinical and nonclinical samples. *Depress. Anxiety* 24 331–341. 10.1002/da.20210 17041921

[B41] Lovibond S. H. Lovibond P. F. (1995). *Manual for the Depression Anxiety Stress Scales.* Sydney: Psychology Foundation of Australia.

[B42] Mantar A. Yemez B. Alkin T. (2010). The validity and reliability of the turkish version of the anxiety sensitivity index-3. *Turk. J. Psychiatry* 21 225–234. 20818510

[B43] Marshall G. N. Miles J. N. V. Stewart S. H. (2010). Anxiety sensitivity and PTSD symptom severity are reciprocally related: evidence from a longitudinal study of physical trauma survivors. *J. Abnorm. Psychol.* 119 143–150. 10.1037/a0018009 20141251 PMC2820125

[B44] McNally R. J. (2002). Anxiety sensitivity and panic disorder. *Biol. Psychiatry* 52 938–946. 10.1016/S0006-3223(02)01475-0 12437935

[B45] Meyer T. J. Miller M. L. Metzger R. L. Borkovec T. D. (1990). Development and validation of the penn state worry questionnaire. *Behav. Res. Ther.* 28 487–495. 10.1016/0005-7967(90)90135-6 2076086

[B46] Mohlman J. Zinbarg R. E. (2000). The structure and correlates of anxiety sensitivity in older adults. *Psychol. Assess.* 12 440–446. 10.1037/1040-3590.12.4.440 11147114

[B47] Morani S. Pricci D. Sanavio E. (1999). Penn state worry questionnaire e worry domains questionnaire. Presentazione delle versioni italiane ed analisi della fedeltà. *Psicoter. Cogn. E Comportamentale* 5 195–209.

[B48] Muthén L. K. Muthén B. O. (2010). *Mplus User’s Guide* 4th Edn Los Angeles, CA: Author.

[B49] Naragon-Gainey K. (2010). Meta-analysis of the relations of anxiety sensitivity to the depressive and anxiety disorders. *Psychol. Bull.* 136 128–150. 10.1037/a0018055 20063929

[B50] Norton G. R. Cox B. J. Hewitt P. L. McLeod L. (1997). Personality factors associated with generalized and non-generalized social anxiety. *Pers. Individ. Dif.* 22 655–660. 10.1016/S0191-8869(96)00243-7

[B51] Noyes R. Carney C. P. Langbehn D. R. (2004). Specific phobia of illness: search for a new subtype. *J. Anxiety Disord.* 18 531–545. 10.1016/S0887-6185(03)00041-0 15149712

[B52] Nunnally J. C. (1978). *Psychometric Theory.* New York, NY: McGraw-Hill.

[B53] Olatunji B. O. Sawchuk C. N. Deacon B. J. Tolin D. F. Lilienfeld S. O. Williams N. L. (2005). The anxiety sensitivity profile revisited: factor structure and psychometric properties in two nonclinical samples. *J. Anxiety Disord.* 19 603–625. 10.1016/j.janxdis.2004.08.005 15927776

[B54] Olatunji B. O. Wolitzky-Taylor K. B. (2009). Anxiety sensitivity and the anxiety disorders: a meta-analytic review and synthesis. *Psychol. Bull.* 135 974–999. 10.1037/a0017428 19883144

[B55] Olthuis J. V. Watt M. C. Stewart S. H. (2014). Anxiety sensitivity index (ASI-3) subscales predict unique variance in anxiety and depressive symptoms. *J. Anxiety Disord.* 28 115–124. 10.1016/j.janxdis.2013.04.009 23770119

[B56] Osman A. Gutierrez P. M. Smith K. Fang Q. Lozano G. Devine A. (2010). The anxiety sensitivity index–3: analyses of dimensions, reliability estimates, and correlates in nonclinical samples. *J. Pers. Assess.* 92 45–52. 10.1080/00223890903379332 20013455

[B57] Otto M. W. Pollack M. H. Fava M. Uccello R. Rosenbaum J. F. (1995). Elevated anxiety sensitivity index scores in patients with major depression: correlates and changes with antidepressant treatment. *J. Anxiety Disord.* 9 117–123. 10.1016/0887-6185(94)00035-2

[B58] Peterson R. A. Heilbronner R. L. (1987). The anxiety sensitivity index: construct validity and factor analytic structure. *J. Anxiety Disord.* 1 117–121. 10.1016/0887-6185(87)90002-8

[B59] Peterson R. A. Plehn K. (1999). “Measuring anxiety sensitivity,” in *Anxiety Sensitivity: Theory, Research, and Treatment in the Fear of Anxiety* ed. Taylor S. (Mahwah, NJ: Lawrence Erlbaum Associates) 61–68.

[B60] Peterson R. A. Reiss S. (1992). *Anxiety Sensitivity Index Manual* 2nd Edn Worthington, OH: International Diagnostic Services.

[B61] R Development Core Team (2013). *R: A Language and Environment for Statistical Computing.* Available at: http://www.R-project.org/

[B62] Rector N. A. Szacun-Shimizu K. Leybman M. (2007). Anxiety sensitivity within the anxiety disorders: disorder-specific sensitivities and depression comorbidity. *Behav. Res. Ther.* 45 1967–1975. 10.1016/j.brat.2006.09.017 17084380

[B63] Reiss S. (1991). Expectancy model of fear, anxiety, and panic. *Clin. Psychol. Rev.* 11 141–153. 10.1016/0272-7358(91)90092-9

[B64] Reiss S. McNally R. J. (1985). “The expectancy model of fear,” in *Theoretical Issues in Behavior Therapy* eds Reiss S. Bootzin R. R. (New York, NY: Academic Press) 107–121.

[B65] Reise S. P. Moore T. M. Haviland M. G. (2010). Bifactor models and rotations: exploring the extent to which multidimensional data yield univocal scale scores. *J. Pers. Assess.* 92 544–559. 10.1080/00223891.2010.496477 20954056 PMC2981404

[B66] Reiss S. Peterson R. A. Gursky D. M. McNally R. J. (1986). Anxiety sensitivity, anxiety frequency and the prediction of fearfulness. *Behav. Res. Ther.* 24 1–8. 10.1016/0005-7967(86)90143-9 3947307

[B67] Rhemtulla M. Brosseau-Liard P. É. Savalei V. (2012). When can categorical variables be treated as continuous? A comparison of robust continuous and categorical SEM estimation methods under suboptimal conditions. *Psychol. Methods* 17 354–373. 10.1037/a0029315 22799625

[B68] Rosseel Y. (2012). lavaan: an r package for structural equation modeling - google scholar. *J. Stat. Softw.* 48 1–36. 10.18637/jss.v048.i02

[B69] Sandin B. Chorot P. McNally R. J. (1996). Validation of the spanish version of the anxiety sensitivity index in a clinical sample. *Behav. Res. Ther.* 34 283–290. 8881099 10.1016/0005-7967(95)00074-7

[B70] Sandin B. García R. M. V. Chorot P. Germán M. A. S. (2007). ASI-3: nueva escala para la evaluación de la sensibilidad a la ansiedad. *Rev. Psicopatol. Psicol. Clín.* 12 91–104.

[B71] Satorra A. (2000). “Scaled and adjusted restricted tests in multi-sample analysis of moment structures,” in *Innovations in Multivariate Statistical Analysis Advanced Studies in Theoretical and Applied Econometrics* eds Heijmans R. D. H. Pollock D. S. G. Satorra A. (New York, NY: Springer) 233–247.

[B72] Scher C. D. Stein M. B. (2003). Developmental antecedents of anxiety sensitivity. *J. Anxiety Disord.* 17 253–269. 10.1016/S0887-6185(02)00202-5 12727121

[B73] Schermelleh-Engel K. Moosbrugger H. Müller H. (2003). Evaluating the fit of structural equation models: tests of significance and descriptivegoodness-of-fitmeasures. *Methods Psychol. Res. Online* 8 23–74.

[B74] Schmidt N. B. Keough M. E. Mitchell M. A. Reynolds E. K. MacPherson L. Zvolensky M. J. (2010). Anxiety sensitivity: prospective prediction of anxiety among early adolescents. *J. Anxiety Disord.* 24 503–508. 10.1016/j.janxdis.2010.03.007 20399075 PMC2872504

[B75] Schmidt N. B. Keough M. E. Timpano K. R. Richey J. A. (2008). Anxiety sensitivity profile: predictive and incremental validity. *J. Anxiety Disord.* 22 1180–1189. 10.1016/j.janxdis.2007.12.003 18242951 PMC2600663

[B76] Schmidt N. B. Zvolensky M. J. Maner J. K. (2006). Anxiety sensitivity: prospective prediction of panic attacks and Axis I pathology. *J. Psychiatr. Res.* 40 691–699. 10.1016/j.jpsychires.2006.07.009 16956622

[B77] Sica C. Coradeschi D. Ghisi M. Sanavio E. (2006). *Beck Anxiety Inventory. Manuale.* Firenze: Organizzazioni Speciali.

[B78] Sica C. Ghisi M. (2007). “The italian versions of the beck anxiety inventory and the beck depression inventory-ii: psychometric properties and discriminant power,” in *Leading-Edge Psychological Tests and Testing* ed. Lange M. A. (Hauppauge, NY: Nova Science Publishers) 27–50.

[B79] Silverman W. K. Goedhart A. W. Barrett P. Turner C. (2003). The facets of anxiety sensitivity represented in the childhood anxiety sensitivity index: confirmatory analyses of factor models from past studies. *J. Abnorm. Psychol.* 112 364–374. 10.1037/0021-843X.112.3.364 12943015

[B80] Simmons J. P. Nelson L. D. Simonsohn U. (2011). False-positive psychology undisclosed flexibility in data collection and analysis allows presenting anything as significant. *Psychol. Sci.* 22 1359–1366. 10.1177/0956797611417632 22006061

[B81] Stein M. B. Jang K. L. Livesley W. J. (1999). Heritability of anxiety sensitivity: a twin study. *Am. J. Psychiatry* 156 246–251. 10.1176/ajp.156.2.246 9989561

[B82] Stewart S. H. Samoluk S. B. MacDonald A. B. Taylor S. (1999). “Anxiety sensitivity and substance use and abuse,” in *Anxiety Sensitivity: Theory, Research and Treatment of the Fear of Anxiety* ed. Taylor S. (Mahwah, NJ: Lawrence Erlbaum) 287–319.

[B83] Stewart S. H. Taylor S. Jang K. L. Cox B. J. Watt M. C. Fedoroff I. C. (2001). Causal modeling of relations among learning history, anxiety sensitivity, and panic attacks. *Behav. Res. Ther.* 39 443–456. 10.1016/S0005-7967(00)00023-1 11280342

[B84] Taylor S. (1999). *Anxiety Sensitivity: Theory, Research, and Treatment of the Fear of Anxiety.* Mahwah, NJ: Lawrence Erlbaum.

[B85] Taylor S. Cox B. J. (1998a). An expanded anxiety sensitivity index: evidence for a hierarchic structure in a clinical sample. *J. Anxiety Disord.* 12 463–483. 10.1016/S0887-6185(98)00028-0 9801964

[B86] Taylor S. Cox B. J. (1998b). Anxiety sensitivity: multiple dimensions and hierarchic structure. *Behav. Res. Ther.* 36 37–51. 10.1016/S0005-7967(97)00071-5 9613015

[B87] Taylor S. Jang K. L. Stewart S. H. Stein M. B. (2008). Etiology of the dimensions of anxiety sensitivity: a behavioral–genetic analysis. *J. Anxiety Disord.* 22 899–914. 10.1016/j.janxdis.2007.09.005 18029140

[B88] Taylor S. Koch W. J. Crockett D. J. (1991). Anxiety sensitivity, trait anxiety, and the anxiety disorders. *J. Anxiety Disord.* 5 293–311. 10.1016/0887-6185(91)90030-W

[B89] Taylor S. Koch W. J. McNally R. J. Crockett D. J. (1992). Conceptualizations of anxiety sensitivity. *Psychol. Assess.* 4 245–250. 10.1037/1040-3590.4.2.245

[B90] Taylor S. Koch W. J. Woody S. McLean P. (1996). Anxiety sensitivity and depression: how are they related? *J. Abnorm. Psychol.* 105 474–479. 10.1037/0021-843X.105.3.474 8772020

[B91] Taylor S. Zvolensky M. J. Cox B. J. Deacon B. Heimberg R. G. Ledley D. R. (2007). Robust dimensions of anxiety sensitivity: development and initial validation of the anxiety sensitivity index-3. *Psychol. Assess.* 19 176–188. 10.1037/1040-3590.19.2.176 17563199

[B92] Telch M. J. Shermis M. D. Lucas J. A. (1989). Anxiety sensitivity: unitary personality trait or domain-specific appraisals? *J. Anxiety Disord.* 3 25–32. 10.1016/0887-6185(89)90026-1

[B93] Van Widenfelt B. M. Treffers P. D. A. Beurs E. de Siebelink B. M. Koudijs E. (2005). Translation and cross-cultural adaptation of assessment instruments used in psychological research with children and families. *Clin. Child Fam. Psychol. Rev.* 8 135–147. 10.1007/s10567-005-4752-1 15981582

[B94] Wardle J. Ahmad T. Hayward P. (1990). Anxiety sensitivity in agoraphobia. *J. Anxiety Disord.* 4 325–333. 10.1016/0887-6185(90)90029-9

[B95] Watt M. C. Stewart S. H. (2000). Anxiety sensitivity mediates the relationships between childhood learning experiences and elevated hypochondriacal concerns in young adulthood. *J. Psychosom. Res.* 49 107–118. 10.1016/S0022-3999(00)00097-0 11068054

[B96] Wheaton M. G. Deacon B. J. McGrath P. B. Berman N. C. Abramowitz J. S. (2012). Dimensions of anxiety sensitivity in the anxiety disorders: evaluation of the ASI-3. *J. Anxiety Disord.* 26 401–408. 10.1016/j.janxdis.2012.01.002 22306133

[B97] White K. S. Brown T. A. Somers T. J. Barlow D. H. (2006). Avoidance behavior in panic disorder: the moderating influence of perceived control. *Behav. Res. Ther.* 44 147–157. 10.1016/j.brat.2005.07.009 16300725

[B98] Won H. T. Park H. S. Kwon S. M. (1995). A study on the development of the Korean versions of Panic Scales. *Korean J. Clin. Psychol.* 14 95–110.

[B99] Zinbarg R. E. Barlow D. H. Brown T. A. (1997). Hierarchical structure and general factor saturation of the anxiety sensitivity index: evidence and implications. *Psychol. Assess.* 9 277–284. 10.1037/1040-3590.9.3.277

[B100] Zvolensky M. J. Arrindell W. A. Taylor S. Bouvard M. Cox B. J. Stewart S. H. (2003). Anxiety sensitivity in six countries. *Behav. Res. Ther.* 41 841–859. 10.1016/S0005-7967(02)00187-0 12781249

[B101] Zvolensky M. J. Feldner M. T. Eifert G. H. Stewart S. H. (2001). Evaluating differential predictions of emotional reactivity during repeated 20% carbon dioxide-enriched air challenge. *Cogn. Emot.* 15 767–786. 10.1080/02699930143000284

[B102] Zvolensky M. J. Forsyth J. P. (2002). Anxiety sensitivity dimensions in the prediction of body vigilance and emotional avoidance. *Cogn. Ther. Res.* 26 449–460. 10.1023/A:1016223716132

